# Bio-Inspired Design of Superconducting Spiking Neuron and Synapse

**DOI:** 10.3390/nano13142101

**Published:** 2023-07-19

**Authors:** Andrey E. Schegolev, Nikolay V. Klenov, Georgy I. Gubochkin, Mikhail Yu. Kupriyanov, Igor I. Soloviev

**Affiliations:** 1 Skobeltsyn Institute of Nuclear Physics, Lomonosov Moscow State University, 119991 Moscow, Russia; tanuior@gmail.com (A.E.S.); mkupr@pn.sinp.msu.ru (M.Y.K.); 2 Faculty of Physics, Moscow State University, 119991 Moscow, Russia; nvklenov@mail.ru (N.V.K.); gubochkin.gi19@physics.msu.ru (G.I.G.); 3 Faculty of Physics, Lobachevsky State University of Nizhny Novgorod, 603950 Nizhny Novgorod, Russia; 4 Russian Quantum Center, 100 Novaya Street, Skolkovo, 143025 Moscow, Russia

**Keywords:** bio-inspired neuron, spiking neural networks, neuromorphic systems, synapse plasticity, Josephson effect, superconductivity

## Abstract

The imitative modelling of processes in the brain of living beings is an ambitious task. However, advances in the complexity of existing hardware brain models are limited by their low speed and high energy consumption. A superconducting circuit with Josephson junctions closely mimics the neuronal membrane with channels involved in the operation of the sodium-potassium pump. The dynamic processes in such a system are characterised by a duration of picoseconds and an energy level of attojoules. In this work, two superconducting models of a biological neuron are studied. New modes of their operation are identified, including the so-called bursting mode, which plays an important role in biological neural networks. The possibility of switching between different modes in situ is shown, providing the possibility of dynamic control of the system. A synaptic connection that mimics the short-term potentiation of a biological synapse is developed and demonstrated. Finally, the simplest two-neuron chain comprising the proposed bio-inspired components is simulated, and the prospects of superconducting hardware biosimilars are briefly discussed.

## 1. Introduction

The cybernetic brain is an old dream of science fiction writers, the embodiment of artificial consciousness created in the image and likeness of man. Despite the remarkable progress of neuroscience in the understanding of the structure and principles of the human brain [1,2,3,4,5,6,7,8,9,10,11] and the improvement of technology and the development of the physical sciences, the cybernetic brain is still science fiction, although this fiction is closer to reality than it was half a century ago.

Inspired attempts to make this dream come true have resulted in a number of steps towards its realisation, such as the development of synaptic chips by large international companies (IBM [12], Google [13], Intel [14] and Qualcomm [15]) as well as by leading scientific organisations [16,17,18]; the creation of auditory and visual implants to restore hearing and vision [19,20,21,22,23,24]; the development of machine learning methods [25,26,27,28,29,30,31,32], including methods that enable people who have lost the ability to move or speak to communicate their thoughts using the brain–computer interface [33]; and, of course, the discovery of the hidden mechanisms of the human brain [34,35,36,37].

Spiking neural networks (SNNs) are a recognised class of neural models that most closely mimic the biological activity of nerve tissue, encoding information by spike sequences, allowing synaptic plasticity and learning [38,39,40]. SNN implementation is in high demand, both for the high-speed simulation of biological neural activity in living tissue, and for real-time systems needed to create and improve bi-directional brain–computer interfaces [33,41].

There are several hardware implementations of bio-inspired SNN. Neuromorphic chips based on silicon or silicon nitride can achieve greater processing power and problem-solving efficiency than general-purpose processors. In addition, semiconductor technology is well understood, affordable, and has a well-developed technology base and convenient interfaces with external devices [41,42]. However, such a traditional approach results in a large number of transistors in the model (realistic from a biological point of view), relatively low performance and high power consumption. Memristors have properties suitable for mimicking biological synapses but need to be coupled [16,43,44] to spiking neurons based on CMOS circuits, and therefore the whole system suffers from the problems described above. The methods of integrated photonics, which have become widespread lately, provide elements with sub-nanosecond characteristic time scales and greater energy efficiency than their semiconductor counterparts [45,46]. Superconducting technologies can be used to build the fastest and most energy-efficient analogue of a biological neuron. A simple superconducting ring, which does not conduct a magnetic field due to the Meissner–Oxenfeld effect, can play the role of a cell membrane. Weak places in the ring (Josephson junctions, JJs), through which magnetic flux quanta can pass, act as analogues of channels for the entry/exit of ions in the operation of a sodium–potassium pump. The time dynamics of the voltage across the Josephson junction during the passage of a quantum is very similar to the typical action potential of a bio-neuron. However, the characteristic time scale here is picoseconds, and the energy dissipated is a fraction of aJ [47,48,49,50,51,52,53,54,55,56,57,58,59]. An additional advantage of the superconducting platform is the possibility to investigate the peculiarities of “quantum” decision making [60] by utilising proven quantum-computing systems [61].

**Figure 1:**
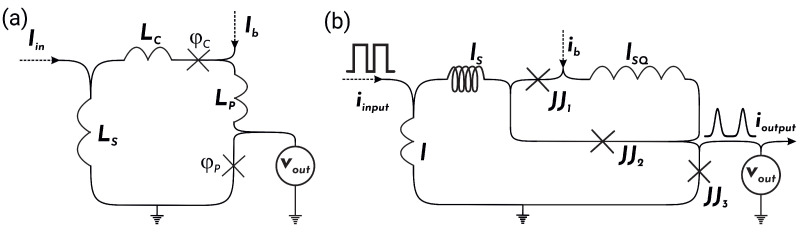
(**a**) Circuit diagram of 2JJ neuron from the work [47] and (**b**) schematic representation of modified spiking neuron (3JJ neuron).

In this article, we study the dynamic processes in the simplest Josephson bio-inspired neuron with two Josephson junctions (2JJ, see Figure 1a) [47] and identify new operation modes that have biological analogues. In particular, we focus on the bursting mode. For the modified bio-inspired neuron with three Josephson junctions (3JJ, see Figure 1b) [62], we reveal the possibility of switching between operation modes in situ on chip. Finally, we propose a modification of synaptic connections that allows synaptic plasticity, an analogue of short-term potentiation (STP). Numerical simulations confirm the validity of the proposed solutions.

## 2. 2*JJ* and 3*JJ* Neurons

### 2.1. Research Methods

The modelling of all systems considered below: 2JJ and 3JJ neurons; axons and different synapses were based on the solution of systems of differential equations for Josephson phases in the frame of the resistively shunted junction (RSJ) model [63]. The state of a Josephson junction is determined by its phase, which serves as a generalised coordinate for the considered nonlinear oscillatory system. The phase time derivative is the voltage normalised with respect to the Josephson junction characteristic value $V_{C}$. The fall of this phase across an inductor corresponds to the magnetic flux normalised with respect to the value of the magnetic flux quantum, $\Phi_{0}$. The input (and output) of magnetic flux quantum into a “superconducting cell” is accompanied by the appearance of a biosimilar voltage pulse (spike) at the Josephson junction.

The equations for the 2JJ neuron, shown in Figure 1a, are as follows:

(1) $$\left\{\begin{array}{c} i_{JJ_{c}}=i_{in}\cdot \Lambda_{s}-\lambda \left(\phi_{c}+\phi_{p}\right)-i_{b}\cdot \Lambda_{p},\\ i_{JJ_{p}}=i_{in}\cdot \Lambda_{s}-\lambda \left(\phi_{c}+\phi_{p}\right)-i_{b}\cdot \left(1-\Lambda_{p}\right). \end{array}\right.$$

Here, $i_{JJ_{c,p}}$ are currents flowing through the “control” ($JJ_{c}$) and “pulse” ($JJ_{p}$) Josephson junctions, having the phases $\phi_{c}$ and $\phi_{p}$, respectively; $i_{in}$ is an input current for the 2JJ neuron; $\lambda ={\left(l_{s}+l_{c}+l_{p}\right)}^{-1}$, where $l_{s,p,c}=2\pi L_{s,p,c}I_{C}/\Phi_{0}$; $\Lambda_{s,p}=l_{s,p}\cdot \lambda$; and $i_{b}$ is a bias current. All currents are normalised by the critical current of the pulse JJ, $I_{CC}$. The time is normalised with respect to the inverse plasma frequency, $\omega_{p}^{2}=2\pi I_{CC}/\Phi_{0}C$, where *C* is a capacitance of the pulse Josephson junction. The currents through the Josephson junctions in the circuit can be expressed as

(2) $$\begin{array}{c} i_{JJ_{p}}={\ddot{\phi }}_{p}+\Gamma {\dot{\phi }}_{p}+\sin\phi_{p}, \end{array}$$

(3) $$\begin{array}{c} i_{JJ_{c}}=\eta \cdot \left({\ddot{\phi }}_{c}+\Gamma {\dot{\phi }}_{c}+\sin\phi_{c}\right), \end{array}$$

where $\Gamma^{2}=\Phi_{0}/2\pi I_{CC}R_{N}^{2}C$ is a damping parameter, and η is a geometric factor reflecting the ratio of the cross-sectional areas of the control and pulse Josephson junctions ($\eta =A_{c}/A_{p}$, the critical current and capacitance of a Josephson junction are assumed to scale linearly with its cross-sectional area).

The equations for the 3JJ neuron, see Figure 1b, are as follows:

(4) $$\left\{\begin{array}{c} i_{JJ_{1}}=-i_{b}-\left(\phi_{1}-\phi_{2}\right)/l_{SQ},\\ i_{JJ_{2}}=i_{b}+\left(\phi_{1}-\phi_{2}\right)/l_{SQ}+i_{in}\cdot \lambda l-\lambda \cdot \left(\phi_{2}+\phi_{3}\right),\\ i_{JJ_{3}}=i_{b}+i_{in}\cdot \lambda l-\lambda \left(\phi_{2}+\phi_{3}\right)-i_{out}, \end{array}\right.$$

where $\lambda ={\left(l+l_{s}\right)}^{-1}$, all inductances ($l,l_{s}$ and $l_{SQ}$) are normalised by $\Phi_{0}/2\pi I_{C}$, $I_{C}$ is the third junction ($JJ_{3}$) critical current, and $i_{out}$ is an output neuron current. In $\omega_{p}$-normalisation, the currents through the Josephson junctions in the circuit can be expressed as follows:

(5) $$\begin{array}{c} i_{JJ_{1}}={\ddot{\phi }}_{1}+\Gamma {\dot{\phi }}_{1}+\sin\phi_{1}, \end{array}$$

(6) $$\begin{array}{c} i_{JJ_{2}}=\eta \cdot \left({\ddot{\phi }}_{2}+\Gamma {\dot{\phi }}_{2}+\sin\phi_{2}\right), \end{array}$$

(7) $$\begin{array}{c} i_{JJ_{3}}={\ddot{\phi }}_{3}+\Gamma {\dot{\phi }}_{3}+\sin\phi_{3}, \end{array}$$

where Γ is also the damping parameter, and η is responsible for the ratio of cross-sectional areas of the 2nd and 3rd Josephson junctions ($\eta =A_{2}/A_{3}$). We also assume that the critical currents of $JJ_{1}$ and $JJ_{3}$ are equal.

### 2.2. Features of the 2JJ and 3JJ Neurons

A detailed analysis of the dynamic processes in terms of the greatest biosimilarity (in the framework of the Hodgkin-Huxley model [64]) even in a simple 2JJ neuron revealed four main regimes or *modes* of operation. These include the *dead mode*, corresponding to a neuron that does not respond to input stimuli; the *injury mode*, where only some of the input stimuli generate a biosimilar response at the output; the *regular mode*, where any standard input stimulus generates a biosimilar voltage response at the output; and the *bursting mode*, where a standard input stimulus leads to the generation of sequential (bursts) of biosimilar pulses [65,66,67,68]. Another regime of operation, the *non-biological mode*, is not the main mode of neuron operation, but it is nevertheless the case for this circuit, characterised by free output pulse generation without any external influence ($I_{in}=0$) under the bias current, $I_{b}$, applied.

From a biophysical point of view, the specific mode of operation is determined by the relationship between the input (corresponding to the $JJ_{c}$) and output (corresponding to the $JJ_{p}$) channel conductances. In our analogy with the sodium–potassium pump, the $JJ_{c}$ junction corresponds to the efflux of $K^{+}$ ions from the cell, while the $JJ_{p}$ junction corresponds to the influx of $Na^{+}$ ions (triggers neuron depolarisation). The higher the critical current of the junction, the lower the corresponding channel conductance. The evolution of processes in the neuron also depends on the bias current that support the “excitation” of the neuron and the parameter Γ, which determines the rate of decay of free current oscillations in the Josephson junctions, as well as the dissipation in the neuron model.

**Figure 2:**
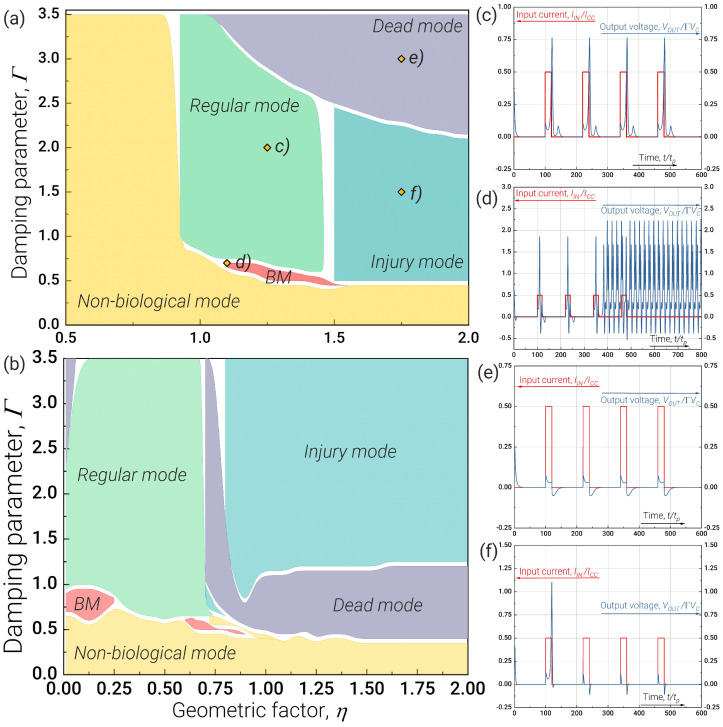
Ranges of parameters for bio-inspired neuron operating modes for damping parameter Γ and geometric factor η for (**a**) 2JJ neuron [47] (see Figure 1a) and (**b**) 3JJ neuron (see Figure 1b). (**c**–**f**) Panels correspond to different operating modes—(**c**) regular, (**d**) bursting, (**e**) dead and (**f**) injury—of the 2JJ neuron (which are qualitatively similar to the operating modes of the 3JJ neuron), marked by yellow diamonds in (**a**). *The non-biological mode* is understood as the dynamics of an artificial neuron that has no analogues in its biological counterpart. The parameters of the 2JJ neuron are as follows: $l_{S}=l_{P}=5$, $i_{b}=1.9$. The parameters of the 3JJ neuron: $l_{S}=3.85$, l=5, $i_{b}=1.9$, $I_{C1}/I_{C3}=1$. The neuron was excited by the input rectangular pulses with level equal to the $A_{input}=0.5$ and pulse duration $\tau =20t_{P}$.

Figure 2a shows that in the 2JJ neuron, all main modes can only be achieved if the geometric factor η is about and above unity. Figure 2c–f illustrate the voltage behaviour at the Josephson junction, simulating the “ion output” for the regular, bursting, dead and injury modes, respectively.

*Regular mode* is a typical neuron cell operation mode with an adequate response to external influence and the proper form of output spikes. Areas corresponding to the regular mode are marked in green (Figure 2) and the typical proper response to the external influence is demonstrated in Figure 2c. The generation of spikes in this regime is associated with the sodium–potassium pump in living nerve cells, altering the ionic balance and the action potential within them. *Dead mode* is also the typical neuron cell operation mode in cases when a nerve tissue is actually damaged, for example, by certain drugs or toxic substances. Such cells are “deaf” to external influences and unable to process signals. Physically, this means no voltage spikes on the pulse Josephson junction when it is externally excited by the current pulses.

*Injury mode* is a transition mode between regular and dead modes, respectively. Injury mode is an incorrect mode of neuron operation when the output does not correspond to the expected regular mode of operation: the neuron responds with only one output spike, whereas it ignores the rest of the external current pulses. *Bursting mode* is a typical and very important mode of neuron cell operation [69,70] characterised by the generation of a series or *bursts* of spikes in response to a single stimulating current pulse. Such behaviour may be the result of the complex interaction of neurons in the network, as well as a consequence of internal processes in a neuron. It plays an important role in synaptic plasticity [65,66], the synchronisation of large groups of neurons [71], the detection of frequency features of input stimuli [72], information coding [73,74] and the reliability of synaptic transmission [65,75], which may be crucial for processing important stimuli [76].

In *non-biological mode*, the level of the bias current $I_{b}$ is sufficient to switch the Josephson junctions of the 2JJ neuron to the resistive state and, as a result, to the persistent generation of spikes. From a biological point of view, such a situation is equivalent to the uncontrolled entry of salt ions from a surrounding medium into a nerve cell. Significantly, at the parameter plane, this mode corresponds to a small attenuation in the system or a violation of the balance between the input and output channels for the magnetic flux quanta (relatively small values of $JJ_{c}$ critical current).

To increase the ability to control the throughput of the input channel, we propose to replace $JJ_{c}$ with a two-Josephson junction superconducting interferometer. Figure 1b shows that this interferometer contains $JJ_{1}$ and $JJ_{2}$. We find all the operating modes described above for the 2JJ neuron for the modified 3JJ neuron as well.

**Figure 3:**
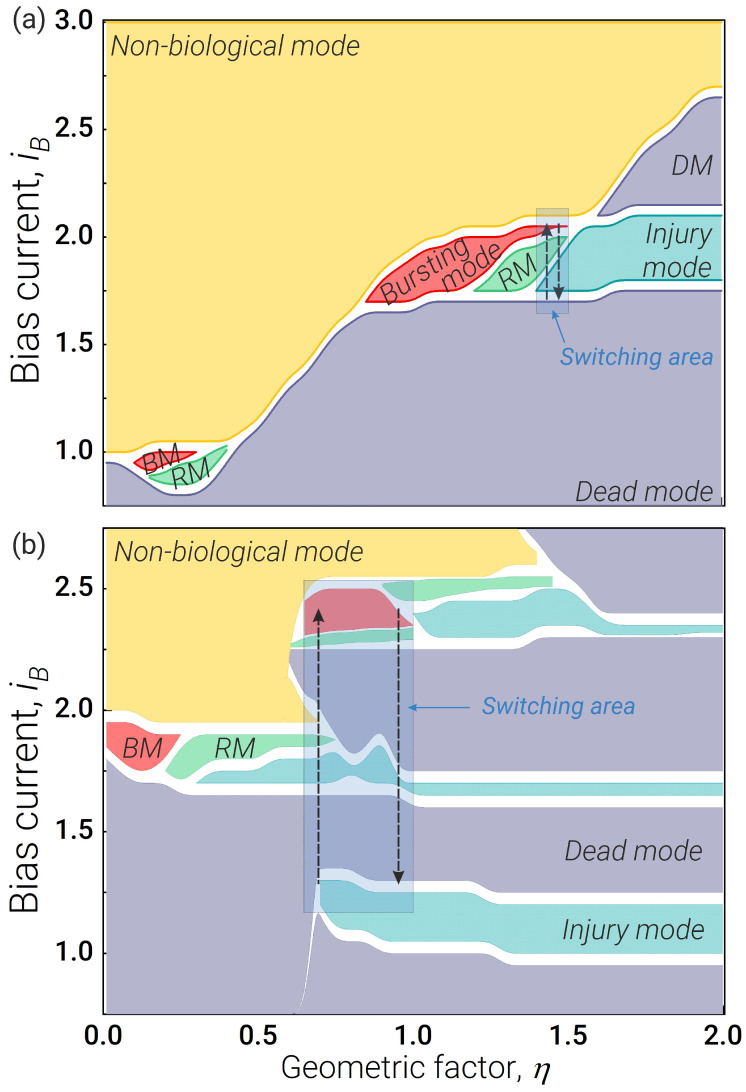
Parameter ranges for the operating modes of the neurons for the bias current $i_{b}$ (which can be adjusted in situ) and the geometric factor η for (**a**) the 2JJ neuron (see Figure 1a) and (**b**) the 3JJ neuron (see Figure 1b). The parameters of the 2JJ neuron are $l_{S}=l_{P}=5$, Γ=0.65. The parameters of the 3JJ neuron are $l_{S}=3.85$, l=5, Γ=0.8. The neuron was excited by rectangular pulses with level $A_{input}=0.5$ and pulse duration $\tau =20t_{P}$. The *switching area* highlighted by blue shows the range in which we can switch between all operating modes by adjusting the bias current $i_{b}$.

The main advantage of the 3JJ neuron is that its operating mode is easier to control. A comparison of Figure 3a,b shows that the 3JJ neuron has a significantly larger parameter range (see light blue areas in figures), in which switching between all operating modes is possible solely by controlling the bias current. By choosing parameters for the 2JJ neuron partly from [47] and partly from Figure 2a ($l_{S}=l_{P}=5$, Γ=0.65) at a fixed value of η, we can switch between all main modes by varying the bias current $i_{b}$ in a narrow range. For the given parameters, this region lies in the range of η from 1.4 to 1.5 and covers only small parts of the bursting and regular mode regions. At the same time, for the 3JJ neuron ($l_{S}=3.85$, l=5, Γ=0.8), we can also switch between all operating modes due to bias current: for the chosen parameters, this range lies in the range of η from 0.65 to 1.0 and covers large parts of the *bursting*, *regular* (twice), *dead* (twice) and *injury* (twice) modes. The appearance of a periodic structure in the parameter plane as a function of $i_{b}$ is associated with the quantisation of the magnetic flux in the circuits of the scheme.

Furthermore, the 3JJ neuron can be made controllable using identical Josephson junctions, and this design tolerates larger variations in physical parameters of the circuit elements. For the technological spread typical of modern digital Josephson circuits [77], we obtain a relatively small offset of the described operating modes on the parameter plane. However, the essence of the phenomena described in the article remains the same.

## 3. Synapse and Axon

Besides the development of a neuron, it is important to provide a mechanism for transferring excitation (action potential) from one neuron to another [78], with the effect of synaptic memory or synaptic plasticity—*short-term potentiation*. External stimulation of the presynaptic neuron should be able to either excite or inhibit the spike activity of the postsynaptic neuron, depending on the synaptic connection between them. The signal transfer through the synapse depends on its history: the more often the excitatory signal comes, the more it is transmitted.

**Figure 4:**
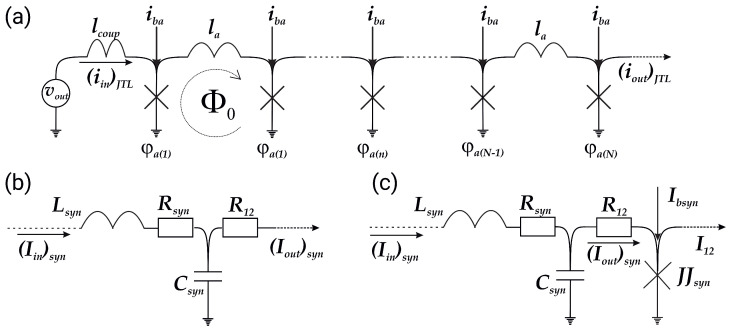
Schematic representation of (**a**) Josephson transmission line and synapses: (**b**) conventional design [47] and (**c**) modified *RLCJ*-synapse with additional Josephson junction connected in parallel to the *RLC*-filter.

The biosimilar model of an axon is a Josephson transmission line (JTL shown schematically in Figure 4a) [79], which is a parallel array of inductively coupled Josephson junctions that transmit spikes corresponding to moving magnetic flux quanta. The equations for the axon JTL have a simple form:

(8) $$\left\{\begin{array}{c} i_{a(1)}=i_{ba}-\frac{\phi_{a(1)}-\phi_{a(2)}}{l_{a}}+{\left(i_{in}\right)}_{JTL},\\ i_{a(2)}=i_{ba}+\frac{\phi_{a(1)}-2\phi_{a(2)}+\phi_{a(3)}}{l_{a}},\\ ...\\ i_{a(n)}=i_{ba}+\frac{\phi_{a(n-1)}-2\phi_{a(n)}+\phi_{a(n+1)}}{l_{a}},\\ ...\\ i_{a(N)}=i_{ba}-\frac{\phi_{a(N-1)}-\phi_{a(N)}}{l_{a}}-{\left(i_{out}\right)}_{JTL}. \end{array}\right.$$

Here, $i_{ba}$ is a bias current applied to each Josephson junction, $\phi_{a(n)}$ is a phase of the *n*-th Josephson junction, and $l_{a}$ is a normalised coupling inductance. The current $i_{a(n)}$ flowing through the *n*-th Josephson junction is

(9) $$i_{a(n)}={\ddot{\phi }}_{a(n)}+\Gamma_{a}{\dot{\phi }}_{a(n)}+\sin\phi_{a(n)},$$

where $\Gamma_{a}$ is the damping parameter. Every JJ in the JTL has a critical current equal to that of the pulse JJ in the neuron. Note that certain myelin sheath properties of biological axons can be modelled by adjusting the value of the coupling inductance $l_{a}$ and parameters of Josephson junctions $JJ_{a(n)}$.

**Figure 5:**
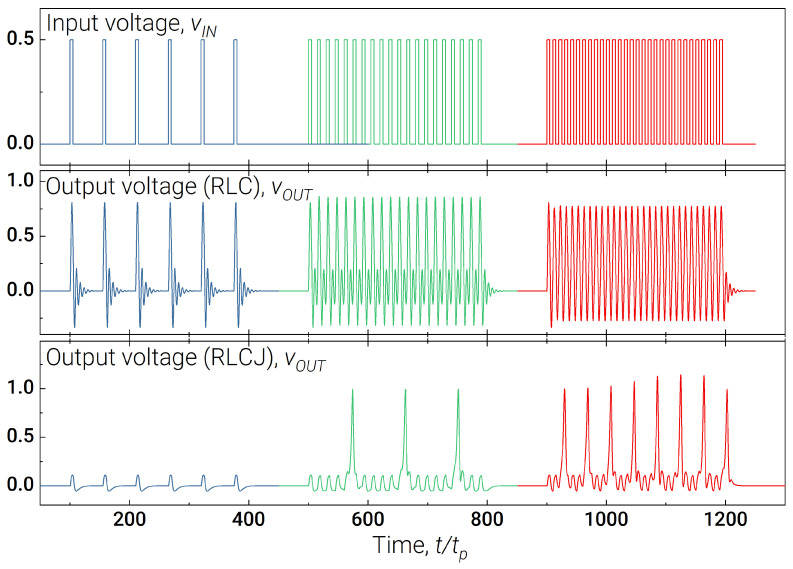
Illustration of one-to-one spike transfer with the conventional RLC synaptic model (proposed in the [47] by P. Crotty et al.) and synaptic plasticity obtained with the modified RLCJ model. The colour of the curves indicates their belonging to the one group consisting of a sequence of input voltage pulses, as well as responses for RLC and RLCJ synapse. The input voltage pulses are the same in both cases and have level $A_{input}=0.5$ and pulse duration $\tau =5t_{p}$. The system parameters are $l_{syn}=2.655$, Q=0.3, $\omega_{0}=1$, $r_{12}=1.2$, $\Gamma_{JJsyn}=\Gamma =1.4$, $i_{bsyn}=0.9$. Both synaptic models were clocked with a series of voltage pulses (the duration of the series was the same each time) with an inter-pulse delay 5$t_{p}$, 10$t_{p}$ and 50$t_{p}$.

A superconducting synapse is usually an RLC-circuit [47], see Figure 4b, where the high-frequency current is grounded through capacitance $C_{syn}$, while resistance $R_{12}$ is used to prevent the bias current outflow from the postsynaptic neuron. The possibility of implementing an excitatory and inhibitory synaptic connection based on such a circuit has already been demonstrated. However, analysis of the amplitude–frequency characteristic shows that it is impossible to model the synaptic plasticity mentioned above using such an RLC filter (see Figure 5).

We propose a modified design of the synapse to capture the short-term potentiation property of its biological counterpart. To suppress the low-frequency current, we add a Josephson junction as shown in Figure 4c. Equations for the obtained *RLCJ* synapse are as follows:

(10) $$\left\{\begin{array}{c} {\ddot{v}}_{syn}=\omega_{0}^{2}\cdot \left\{-\frac{Q}{\omega_{0}}{\dot{v}}_{syn}-v_{syn}+{\left(v_{in}\right)}_{syn}-\left[l_{syn}\cdot \frac{d}{dt}+Q\omega_{0}l_{syn}\right]\cdot {\left(i_{out}\right)}_{syn}\right\},\\ {\left(i_{out}\right)}_{syn}=\frac{\Gamma }{r_{12}}\cdot \left(v_{syn}-v_{JJsyn}\right),\\ v_{JJsyn}={\dot{\phi }}_{JJsyn},\\ {\ddot{\phi }}_{JJsyn}=i_{bsyn}+{\left(i_{out}\right)}_{syn}-i_{12}-\Gamma_{JJsyn}{\dot{\phi }}_{JJsyn}-\sin\phi_{JJsyn},\\ \frac{{\left(i_{out}\right)}_{syn}}{dt}=\frac{\Gamma }{r_{12}}\cdot \left\{{\dot{v}}_{syn}-i_{bsyn}-{\left(i_{out}\right)}_{syn}+i_{12}+\Gamma_{JJsyn}{\dot{\phi }}_{JJsyn}+\sin\phi_{JJsyn}\right\}, \end{array}\right.$$

where the abbreviation $JJ_{syn}$ stands for the synaptic Josephson junction, $JJ_{syn}$; $\omega_{0}$, *Q*, and $l_{syn}$ are the resonant frequency, quality factor and inductance of the RLC-filter, respectively; $r_{12}$ is a coupling resistance between the *RLC* part of the synapse and its Josephson junction; and $i_{bsyn}$ is a synaptic bias current. The resulting *RLCJ* synapse changes the repetition frequency of the passing pulses proportional to this frequency, see Figure 5. The higher the frequency at the input, the greater the number of spikes at the output.

## 4. Two-Neuron Chain

**Figure 6:**
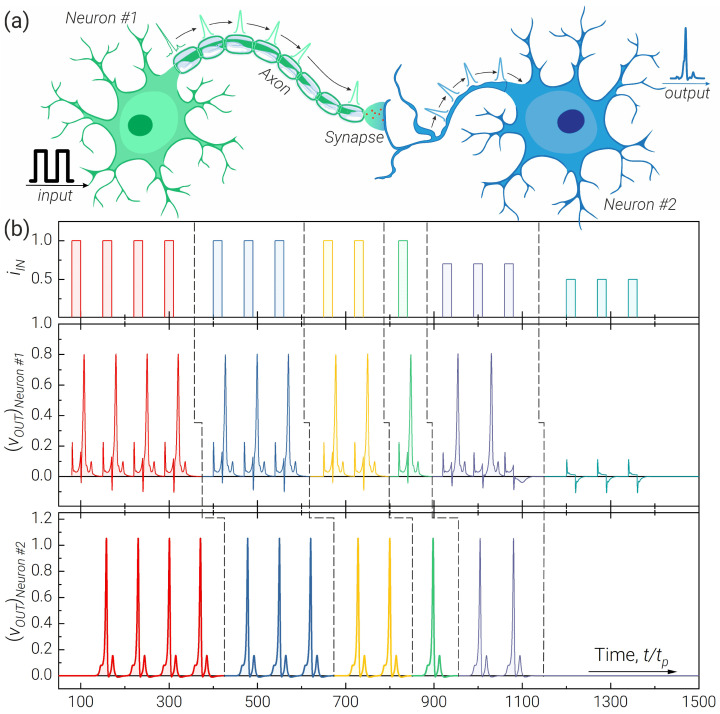
Illustration of a two-neuron chain operation composed of two 3JJ neurons, JTL as an axon, and a *RLCJ*-synapse. (**a**) Biosimilar sketch of the system. (**b**) Output pulses of excitatory (*Neuron #1*) and postsynaptic (*Neuron #2*) neurons for different levels and numbers of the input signals, $i_{in}$. Parameters of the system: l=5, $l_{S}$ = 3.85, $l_{SQ}=l+l_{S}$, $i_{b}=1.9$, Γ=1.4, η=0.2; $l_{coup}=1$, $i_{bA}=0.95$, $l_{A}=0.3l_{SQ}$, $\Gamma_{A}=2$, number of JJ in the JTL =10, $r_{a}=1.2$; $l_{syn}=l_{A}$, $\omega_{0}=1$, Q=0.3, $r_{12}=1.2$, $\Gamma_{JJsyn}=1.4$, $i_{bsyn}=0.9$.

We also simulated two neurons connected through the axon and synapse (see Figure 6a). For this simple network architecture, we used the developed 3JJ-neuron model, a JTL as an axon and a modified *RLCJ* circuit as a synapse. Figure 6b demonstrates the correct operation of the studied circuit in the considered modes. The postsynaptic neuron is connected here to the circuit with the synapse via the resistor $R_{a}$ to eliminate the possibility of parasitic backaction.

From the results of the simulation shown in Figure 6, it is clear that there is potential for the Josephson excitatory neuron to function efficiently. However, in the real brain, inhibitory neurons also play an important role. In order to further develop the research presented, it is necessary to demonstrate the functioning of an inhibitory neuron in a system consisting of at least three neurons (excitatory, inhibitory and target neuron).

## 5. Conclusions

In this work, we explored new bio-inspired modes of operation of a conventional 2JJ superconducting model of a biological neuron. We described the analogy between the operation of the sodium–potassium pump and the dynamics of magnetic flux penetration in a superconducting circuit with Josephson junctions. We showed for the first time that it is possible to switch between the operating modes in situ by adjusting the bias current of the circuit. The use of the modified 3JJ biosimilar neuron provides a significant increase in the parameter margins of the switching area. We also studied the transfer function of the conventional synaptic RLC-model. We proposed its RLCJ modification to mimic the synaptic plasticity, i.e., the short-term potentiation of a biological synapse. Finally, we simulated the simplest two-neuron chain composed of 3JJ neurons, RLCJ-synapse and axons modelled by JTLs. Its correct operation in the considered modes is demonstrated. Optimisation at the architectural level and experimental implementation of the networks modelling neural tissue are the next step in this field of research. Another important direction for extending the applicability of superconducting hardware models is to mimic other types of synaptic plasticity, such as spike-timing-dependent [59] and long-term plasticity. Together with addressing the issue of the down-scaling of superconducting circuits, e.g., by pursuing the inductor-less approach [80,81], these efforts are paving the way for the fast and energy-efficient hardware embodiment of artificial intelligence.

## Data Availability

All relevant data are included in the article.
